# Contamination of wounds with fecal bacteria in immuno-suppressed mice

**DOI:** 10.1038/s41598-020-68323-5

**Published:** 2020-07-13

**Authors:** Lisa Karner, Susanne Drechsler, Magdalena Metzger, Paul Slezak, Johannes Zipperle, Guadalupe Pinar, Katja Sterflinger, Friedrich Leisch, Johannes Grillari, Marcin Osuchowski, Peter Dungel

**Affiliations:** 1 Ludwig Boltzmann Institute for Experimental and Clinical Traumatology, AUVA Research Center, Donaueschingenstraße 13, 1200 Vienna, Austria; 2 Department of Biotechnology, University of Natural Resources and Life Sciences, Vienna, Austria; 3 Institute of Statistics, University of Natural Resources and Life Sciences, Vienna, Austria

**Keywords:** Health care, Medical research

## Abstract

Immunocompromised patients are predisposed to chronically infected wounds. Especially ulcers in the dorsal region often experience secondary polymicrobial infections. However, current wound infection models mostly use single-strain bacteria. To mimic clinically occurring infections caused by fecal contamination in immunocompromised/immobile patients, which differ significantly from single-strain infections, the present study aimed at the establishment of a new mouse model using infection by fecal bacteria. Dorsal circular excision wounds in immunosuppressed mice were infected with fecal slurry solution in several dilutions up to 1:8,000. Impact of immunosuppressor, bacterial load and timing on development of wound infections was investigated. Wounds were analyzed by scoring, 3D imaging and swab analyses. Autofluorescence imaging was not successful. Dose-finding of cyclophosphamide-induced immunosuppression was necessary for establishment of bacterial wound infections. Infection with fecal slurry diluted 1:166 to 1:400 induced significantly delayed wound healing (p < 0.05) without systemic reactions. Swab analyses post-infection matched the initial polymicrobial suspension. The customized wound score confirmed significant differences between the groups (p < 0.05). Here we report the establishment of a simple, new mouse model for clinically occurring wound infections by fecal bacteria and the evaluation of appropriate wound analysis methods. In the future, this model will provide a suitable tool for the investigation of complex microbiological interactions and evaluation of new therapeutic approaches.

## Introduction

Each year, 305 million people suffer from acute, traumatic or burn wounds, globally^1^. The European community spends 2–4% of the total health expenditure on wound treatment^2^. Wounds can arise from injuries, surgeries and as a consequence of extrinsic factors and underlying comorbidities (e.g. vascular diseases or diabetes). Hence, the general classifications differentiate between acute (e.g. burns or surgical wounds) and chronic wounds, (e.g. vascular, diabetic or pressure ulcers). In healthy individuals, the acute wounds generally heal without complications with basic supportive care and minimal infection risk. However, comorbidities and/or risk factors such as vascular diseases, diabetes, a weak immune system, bacterial colonization (especially with pathogens of high intrinsic virulence/resistance) may frequently lead to the development of a chronically infected wounds. Skin and soft tissue infections (SSTIs) represent the most common infections in humans^3^. The associated impairment of wound healing incurs financial and logistic burden to the health care system and lowers the quality of life in affected patients^4^.

In the clinical setting, secondary infections of chronically non-healing wounds further aggravate their complex pathology and delay the healing processes. Immunocompromised patients are prone to develop chronically infected wounds^5^, and especially patients with pressure ulcers often experience secondary polymicrobial infections. Ulcer wounds typically occur in the coccyx region and due to their proximity to the sphincter and increased fecal incontinence in these, by majority elderly patients, they are prone to contamination with intestinal bacteria^6^. In this regard, so-called ESKAPE pathogens (*Enterococcus faecium*, *Staphylococcus aureus*, *Klebsiella pneumoniae*, *Acinetobacter baumannii*, *Pseudomonas aeruginosa*, and *Enterobacter* spp., e.g. *Escherichia coli*) are of particular importance, also, due to their frequent occurrence in nosocomial infections^7,8^.

Wound infection models have been established in several species, with rodents as the most frequently used experimental platform^9,10^. Most often, wounds in these rodent models were induced by skin abrasion^11–16^ or burn injury^17–21^. To recapitulate larger wounds as caused by ulcers and/or traumatic injuries, excision wounds are a more suitable model. Hamblin et al.^22^ used such a model to quantify bacterial single-strand wound infections by bioluminescence imaging^23–25^.

Infections with single, isolated pathogens have been used in the majority of pre-clinical studies, despite the fact that the majority of clinically encountered acute (and also chronic) wound infections are polymicrobial and feature mixed aerobic and anaerobic populations^26^. Preclinical models representing polymicrobial infections are sparse^27–29^, especially using bacteria derived from the same animal species. To fill this gap, here we developed an in vivo model of feces-contaminated wound infection in immunosuppressed mice. Immunosuppression was induced by application of cyclophosphamide, which leads to neutropenia. The model comprises the infection of a dorsal full-thickness excision wound by a topical application of a fecal slurry.

## Methods

### Animals

12 weeks old female BALB/c mice (total n = 85) from Janvier Labs with an average weight of 20–25 g were used for the experiments. Mice were housed in groups of 4 animals per Type-III cage on a 12 h light–dark diurnal cycle with room temperature between 21 and 23 °C. Standard rodent diet (Abbedd Lab & Vet Service, Vienna, Austria) and water were provided ad libitum throughout the experiments. Cages were enriched with carton houses, wooden boards, small blocks for gnawing as well as wood wool for nesting (Abbedd Lab & Vet Service, Vienna, Austria) to facilitate natural behavior prior to and throughout the experiments. Mice were acclimated for 10 days before the experiments.

### Cecal slurry (CS) preparation

Cecal slurry was prepared according to a modified procedure, which was previously described^30^. Briefly, 20 female, 10-weeks old BALB/c mice were sacrificed and their whole ceca dissected. Total cecal content was collected in sterile petri dishes using sterile forceps and spatula and mixed with double distilled water (ddH_2_O) at a ratio of 0.5 ml ddH_2_O per 0.1 g of cecal content. The suspension was strained through both an 800 µm and sequentially a 100 µm sieve (HAVER Test Sieve, VWR International, Radnor, Pennsylvania). The volume of the filtrate was determined and mixed with an equal amount of 30% glycerol solution in phosphate buffered saline (PBS). CS aliquots were stored at − 80 °C until use.

For infection procedure, an aliquot was thawn and centrifuged (16.000 g, 3 min, room temperature, Biofuge Pico, Heraeus, Hanau, Germany). The supernatant was discarded, and the pellet was resuspended in 30 µl of 0.9% sterile saline. This suspension is later indicated as stock solution. The bacteria concentration of the CS stock solution was quantified using the Bacteria Counting Kit, as described below, and showed a concentration of 1 × 10^9^/ml. All dilutions were prepared from this stock by mixture with sterile 0.9% sterile saline.

### Wound model

#### Surgery

To induce temporary neutropenia, mice were pretreated with intraperitoneal cyclophosphamide (CPA) injections of 150 mg/kg on day 4 and 100 mg/kg on day 1 before surgical procedure.^31^ Under inhalation anesthesia the skin was depilated, disinfected (Isozid, Gebro Pharma, Vienna) and a 1 cm circular full-thickness excision wound was cut on the dorsal median line using surgical scissors and forceps.

#### Polymicrobial wound infection

A suspension of 30 µl of CS was used to inoculate the wounds in the infection group. Control animals were treated with 30 µl of 0.9% sterile saline and their wounds were additionally disinfected during each dressing change using Octenisept (Schülke & Mayr GmbH, Norderstedt, Germany).

#### Wound dressing

**Figure 1:**
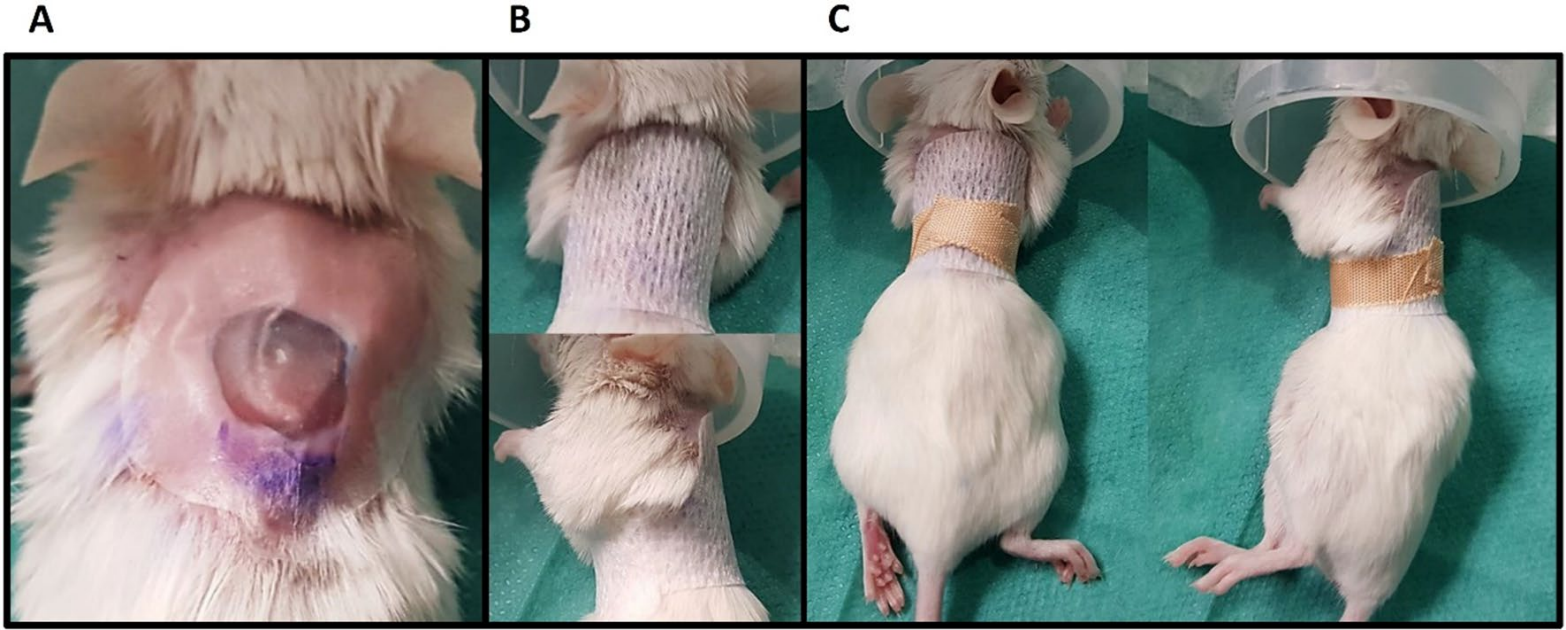
Functional 4-layered wound dressing to protect from external influences and keep a moist environment consisting of **(A)** Nugel hydrogel covered with Suprasorb F transparent film dressing, which is fixed with **(B)** hypafix adhesive bandage and **(C)** leucoplast tape.

In between the procedures, the wounds were covered with a four-layered wound dressing. 0.05 ml hydrogel (Nugel, Systagenix, North Yorkshire, England) were applied directly onto the wound in order to maintain a moist wound bed environment and the wound covered with Suprasorb F transparent film dressing (Lohmann&Rauscher, Germany) (Fig. 1A). This functional bandage was fixed with a two-layered retention bandage (Hypafix and Leucoplast; Fig. 1B, C). Wound dressings were changed on day 1, 3 and 6 post-surgery or whenever they became loose. Dressings were removed on day 7. Wound healing was tracked at least until day 7 and/or up to the day of wound closure.

#### Wound closure analysis

To follow the course of wound closure, digital 3D photos were taken at every wound dressing change using a stereoscopic camera (LifeViz micro, Quantificare, France). Wound area was analyzed by a planimetric measurement with a free-hand tool using Fiji image processing software (ImageJ, National Institute of Health, USA). The percentage of the wound area was calculated using the following formula:

$$Wound area \left[\%\right]= \left[\frac{wound area (day n)}{wound area (day 0)} \right]\times 100$$

where the wound area (day 0) is the area measured directly after surgery and wound area (day n) indicates the area on n days after surgery.

### Quantitative microbiological analysis of CS

Bacteria in the cecal slurry were quantified by flow cytometry using the Bacteria Counting Kit (Thermo Fisher Scientific, Waltham, MA, USA) according to the manufacturer’s instructions. Briefly, one vial of CS was centrifuged, and the pellet was resuspended in 500 µl 0.9% saline (1:16 dilution of the stock solution). The sample was further diluted 1:500 with 0.9% saline (1:8,000 of the stock solution) and 1 ml of this dilution was then stained with 1 µl SYTO BC dye. For quality check, 10 µl of microsphere standard beads (6 µm) were added to a second sample of the same dilution. After 5 min incubation at room temperature in the dark, the samples were analyzed using the flow cytometer (CytoFLEX AS16153, Beckman Coulter, CA, USA). The dye was excited using the laser emitting at 488 nm and the emission was recorded in the fluorescein channel.

### Metagenome analysis of CS via nanopore sequencing technology

The methodology and data analysis was recently described in a paper of Pinar et al.^32^.

#### DNA extraction

Total DNA was isolated from 2 ml of CS. The slurry was vortexed for sample homogenization and then centrifuged (9,279 g, 10 min, room temperature, Centrifuge 5415R, Eppendorf, Hamburg, Germany). The pellet (~ 100 mg) was subjected to DNA extraction using the FastDNA Spin Kit for Soil (MP Biomedicals, Illkirch-Graffenstaden, France) according to manufacturers’ recommendations. To obtain the DNA purity and match the Nanopore workflow’s requirement, the DNA extract was further purified using the QIAamp Viral RNA Mini Kit (Qiagen, Venlo, Nederland). The DNA concentration was assessed by using the Qubit 2.0 Fluorometer with the Qubit dsDNA BR Assay Kit (Invitrogen, Carlsbad, USA).

##### (a) Library construction, template preparation and sequencing

DNA libraries were constructed following the “1D Genomic DNA by Ligation” (SQK-LSK109) available in the Oxford Nanopore community using the Flow Cell Priming Kit EXP-FLP001 (Oxford Nanopore Technologies, Oxford, United Kingdom). All steps for library preparation were performed following the specifications of the protocol using 1 µg input of extracted DNA. Following the preparation of the DNA ends for adapter attachment using the NEBNext FFPE DNA Repair Mix (M6630, New England Biolabs, MA, USA) and the NEBNext End Repair/dA-Tailing Module (E7546 New England Biolabs, MA, USA), the attachment of sequencing adapters (supplied in the kit) to the DNA ends was achieved by means of the NEBNext Quick Ligation Module (E6056, New England Biolabs, MA, USA).

After library preparation was completed, a flow cell quality control (FLO-MIN 106 R9 version, Oxford Nanopore Technologies, Oxford, United Kingdom) was run prior to starting sequencing. The MinKNOW software (Oxford Nanopore Technologies, Oxford, United Kingdom) was used to check the number of active pores in the flow cell. Finally, the priming and the loading of the DNA library into the flow cell were performed according to the recommendations of the manual supplied by the manufacturers.

The sequencing run was set up as follows: the Nanopore device (MinION) was connected to a portable computer and the software MinKNOW was launched after entering information about the sample (i.e. Sample and Flow cell ID) and selecting the appropriate protocol script. Run was performed for 24 h.

##### (b) Data analyses

Resulting fast5 data files were basecalled using the Nanopore GPU basecalling with GUPPY 3.0.3 on UBUNTU 16.04 (Oxford Nanopore Technologies Community Platform, Oxford, United Kingdom). Once the Fastq files were generated, the data was compared with databases using one of the available pipelines for data analyses of the Nanopore Community Platform, following the steps recommended by the manufacturers. The selected workflow chosen was “What's in my pot” (WIMP), which is an EPI2ME workflow for taxonomic classification of basecalled sequences (reads) generated by Nanopore sequencing. WIMP initially filters FASTQ files with a mean q-score below a minimum threshold (defaults to 7). For reads above the quality threshold, the Centrifuge Classification Engine is executed to assign each read to a taxon in the NCBI taxonomy.

### Microbiological swab analysis of the wound bed

A semi-quantitative microbiological swab analysis was conducted to characterize the wound infection. On day 3 post-surgery, a sterile cotton swab (Sterile R, Meus S.R.L., Piove di Sacco, Italy) was used to take a representative microbiological sample from the wound area directly after the wound dressing removal. The samples were analyzed and adjudged by a veterinary diagnostics laboratory (Invitro, Vienna). This analysis included a total cultural differentiation and isolation with a following identification via MALDI-TOF analysis. If necessary, a further biochemical differentiation according to the analytical profile index (API) was performed.

### Wound score

For wound monitoring, a wound score was developed based on clinical observations and the wound closure. Table 1 shows the defined categories and the scoring criteria.

**Table 1:** A custom-made wound score ranging from 0 (best) to 3 (worst) based on clinical observation for wound monitoring.

Score	0	1	2	3
**Category**				
Swelling of wound edge	No	1–33%	34–66%	67–100%
Amount of liquid exudate	Dry	Little	Medium	A lot
Yellow fibrin/pus layer	No	Thin	Medium	Thick
Scab formation	No	On the edges	Thin	Thick
Erythema	No	Slight	Medium	Heavy

At each consecutive wound dressing change the wounds of all mice were evaluated and scored by two independent persons from 0 (best) to 3 (worst) according to Table 1. The swelling of the wound edges, amount of liquid exudate, thickness of a yellow fibrin/pus layer, area covered, and thickness of formed scabs and severity of erythema were graded based on a visual inspection assessment. The sum of the wound scores was compared at different time points within the different experimental groups.

### Autofluorescence imaging

Autofluorescence signals of the full-excision wounds were acquired daily (at dressing changes) using the high-performance multispectral Maestro in vivo imaging system (CRI, Woburn, Massachusetts) at days 1–7 post-surgery. The autofluorescence signal of the CS colonies formed after incubation on LB agar petri dishes were used as the reference signal to select the optimal process settings. Using the blue filter set (M-MSI-FLTR-BLUE, excitation 445–490 nm; emission 515 nm longpass), fluorescence was recorded from 500 to 720 nm in 10 nm steps for an automatically optimized exposure time (ms). The exposure time was kept constant for each mouse for all imaging time points. After linear unmixing of the autofluorescence signal of interest (CS colony) from the one of fur and wound exudate within a defined and constant region of interest (ROI), the total signal in counts/s was analyzed and compared within the groups.

### Statistical analysis and data presentation

All statistical analyses were performed using the statistics software GraphPad (GraphPad Software Inc., Version 5.0, CA, USA) and all data are presented as mean ± SD. P values of ≤ 0.05 were considered significant. The wound closure rates of wounds with and without bandages as well as the wound scores among groups and time points were compared using a two-way ANOVA of grouped analyses with Bonferroni correction to control for type I error in multiple comparisons.

All figures and charts were prepared with Excel 2013 (Microsoft Corporation**,** WA, USA; https://www.microsoft.com) and GraphPad 5.0 (GraphPad Software Inc., CA, USA; https://www.graphpad.com).

### Ethical statement

All procedures were approved by the Animal Protocol Review Board of the City Government of Vienna, Austria (vote: 308358/2018/15) and were in accordance with the National Institute of Health guidelines for the use and care of laboratory animals. To ensure a comprehensive observation, all animals were checked by trained professionals (i.e. DVMs and/or trained personnel) at least 3 times per day once they entered the experiment. Throughout the experiment, all mice received daily analgesic treatment (Meloxicam, 1 mg/kg, sid, s.c. or p.o.). In case of sustained signs of pain, mice were treated with buprenorphine (Bupaq, 0.1 mg/kg tid, s.c, Richter Pharma, Austria). All surgical procedures were done under inhalation anesthesia of 3–6% Sevoflurane (Sevorane, AbbVie Inc., North Chicago, Illinois, USA). Signs of systemic infection persisting for more than 24 h led to euthanasia of the animal based on predefined humane endpoints. At the end of the regular observation period all mice were killed under deep inhalation anesthesia with sevoflurane followed by cervical dislocation.

## Results

### Influence of the wound dressing on the wound

**Figure 2:**
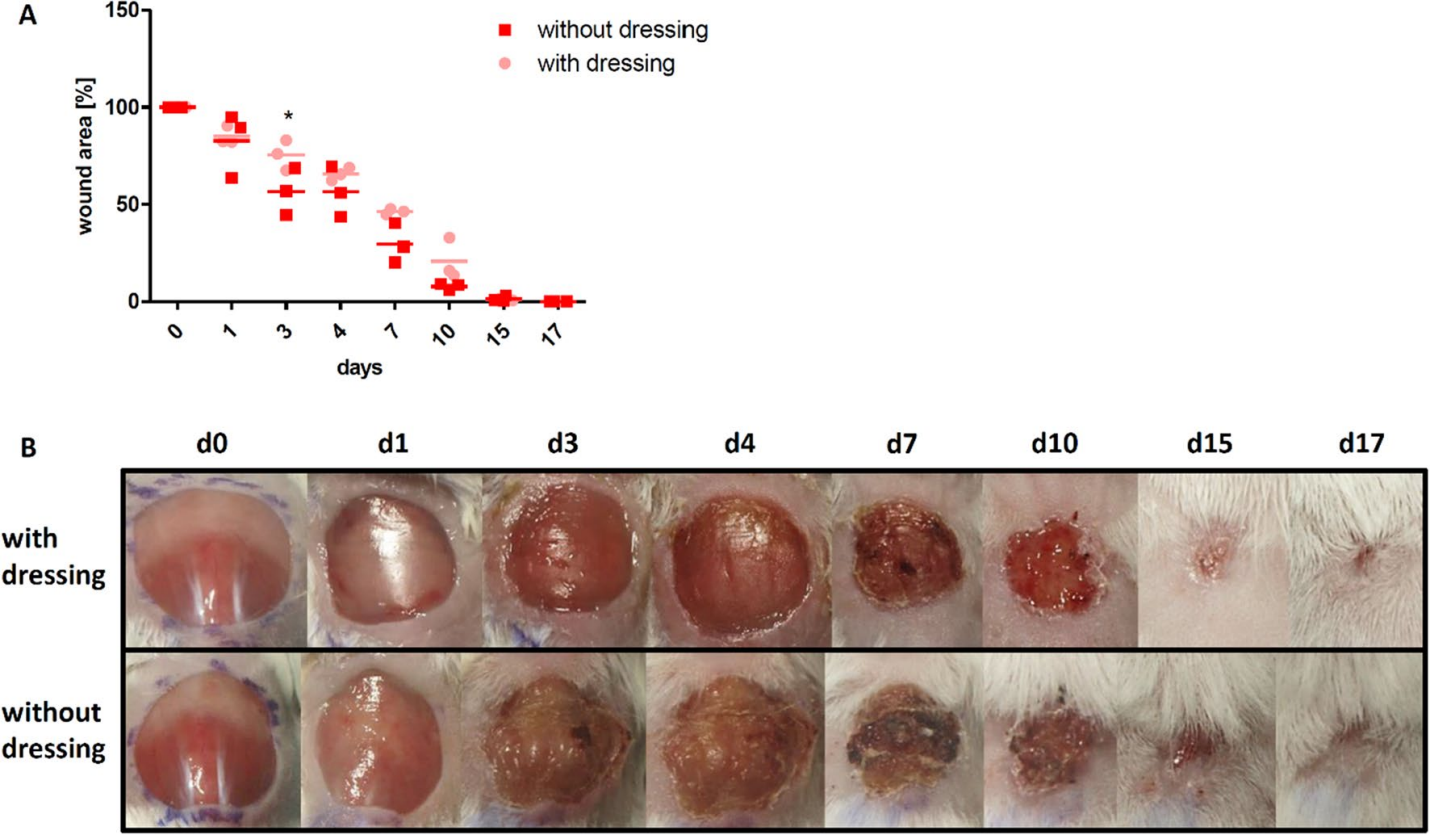
Influence of the wound dressing on the wound closure rate. **(A)** Wound closure rate is slightly faster and less consistent in the group without dressing, showing a significant difference on day 3. **(B)** Exemplary course of wound healing up to day 17 for a mouse with wound dressing and a mouse without wound dressing, showing the strong scab formation in the group without wound dressing. Mean ± SD; * P ≤ 0.05.

Since long-term experiments with mice are very challenging regarding the durability of wound dressings, we tested the necessity to cover wounds. All wounds healed within 17 days irrespective of dressing.Although uncovered wounds seemed to close faster, which was even significant on day 3, the dry wound bed in this group was strongly associated with scab formation (Fig. 2B) and large wound area variations. In contrast, wounds covered with dressing showed a more homogenous wound healing process reflected by a continuously lower variation in wound size, i.e. at least twofold lower variation coefficient in dressed versus uncovered wounds (Fig. 2A). In accordance with the clinical standard procedure for treatment of acutely infected wounds, all subsequent experiments were performed with wound dressing.

### Influence of cyclophosphamide on wound infection and wound closure

**Figure 3:**
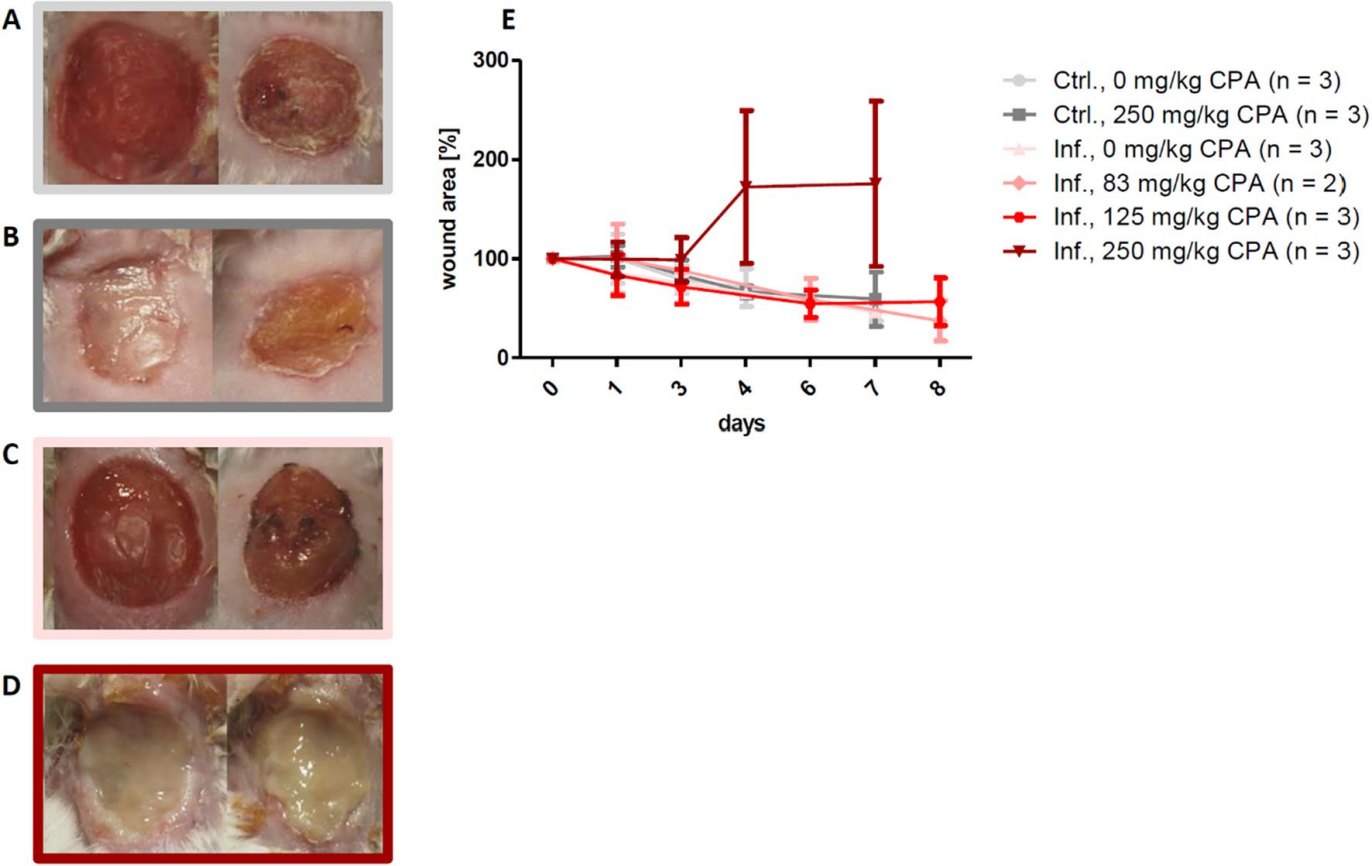
Influence of cyclophosphamide (CPA) induced neutropenia on the formation of wound infections. Exemplary wound pictures on day 5 (left) and day 8 (right) in **(A)** the control group without CPA, **(B)** the control group with 250 mg/kg total dose of CPA, **(C)** the infection group without CPA and **(D)** the infection group with 250 mg/kg total dose of CPA. **(E)** 250 mg/kg CPA was needed to induce delayed wound healing. No pretreatment or reduction of the CPA dose to 83 mg/kg or 125 mg/kg total dose did prevent infection establishment and the wound healing rate was similar to the control group. Mean ± SD.

Cyclophosphamide is a chemotherapeutic agent that induces neutropenia. To produce an environment of an inadequate microbial clearance (i.e. by reducing the neutrophil infiltration) with delayed wound healing, two-time intraperitoneal injection of a total dose of 250 mg/kg CPA was required before surgery/infection (Fig. 3A–E). We investigated a reduced CPA dosing to minimize systemic side effects, while maintaining the healing delay. However, without pretreatment or with lower doses of CPA, i.e. 83 mg/kg or 125 mg/kg, the immune system remained active and bacterial infection was prevented, so that wound healing speed was comparable to control mice without CPA (Fig. 3A–E).

### Influence of CS dose on wound infection and wound closure

**Figure 4:**
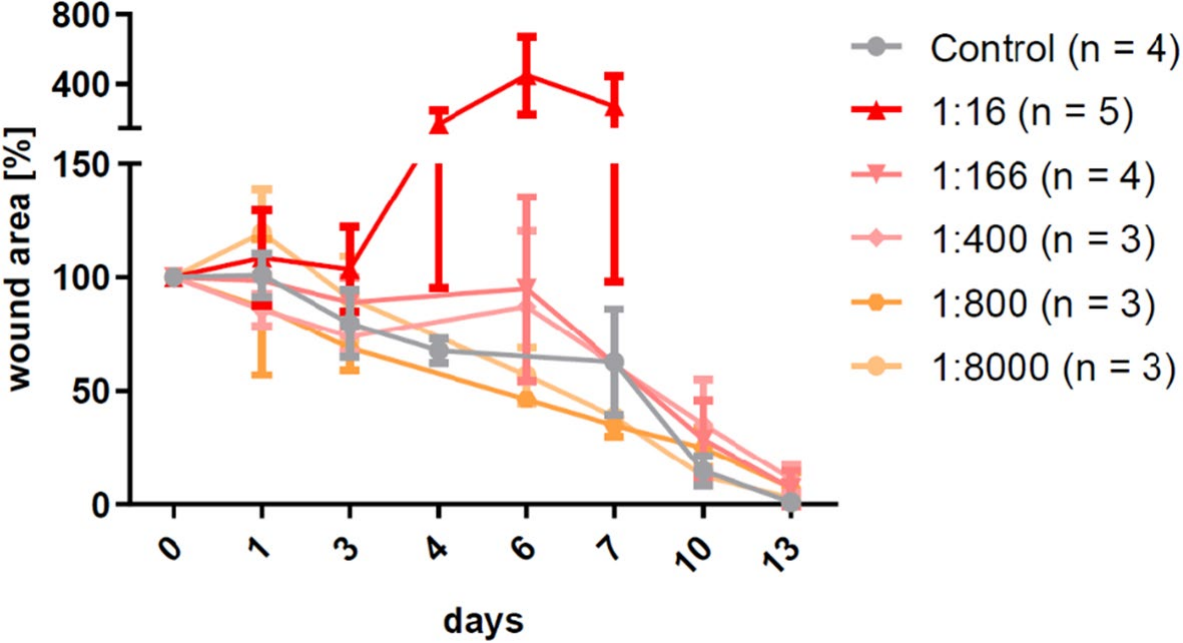
Influence of the cecal slurry (CS) concentration on the establishment of wound infections and the delay of wound healing. Strong systemic reaction rendered the stock concentration (1 × 10^9^/ml bacteria) as well as the 1:16 dilution unsuitable for the model and the 1:800 and 1:8,000 dilutions were not showing any difference compared to the control group. Concentrations between 1:166 and 1:400 (6 × 10^6^ to 2.5 × 10^6^/ml bacteria) contained the highest possible concentration of pathogens that showed a wound healing delay without causing signs of systemic reactions. Mean ± SD.

After verifying the correct CPA dose, several dilutions of CS inoculum were tested to find an optimal CS concentration ensuring a stable wound infection. First pilot studies showed that an inoculum of 30 µl of the undiluted CS stock solution (1 × 10^9^/ml bacteria) or 1:16 diluted stock solution (6.25 × 10^7^/ml bacteria) in mice pretreated with 250 mg/kg CPA resulted in unwanted clinical signs of systemic infection. Based on the predefined humane endpoint criteria these mice were excluded from the experiment. In another setup the dilutions 1:166, 1:400, 1:800 and 1:8,000 of the CS stock were tested (Fig. 4). The 1:166 and 1:400 dilutions (between 6 × 10^6^/ml and 2.5 × 10^6^/ml bacteria) contained the highest possible concentration of pathogens that resulted in comparable, delayed wound closure rates and well-established infections without any signs of systemic effects up to six days after infection.

### Assessment of the established infection

#### Wound score, wound area, and swab analysis

**Figure 5:**
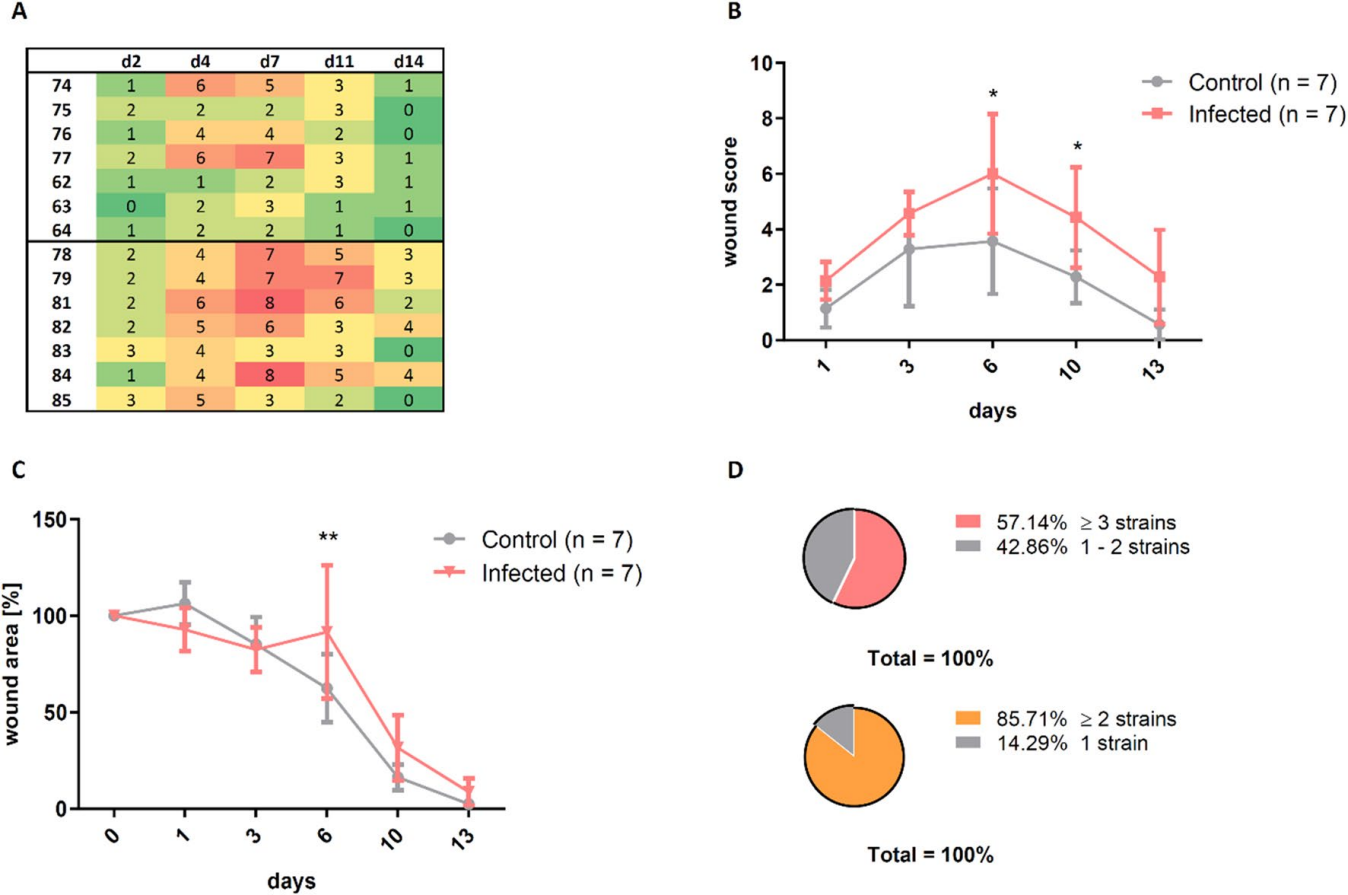
The wound score enabled the differentiation between the control group and the infected group. **(A)** The sum of the assigned scores for all parameters is presented as a heat map, where green shows a good and red a bad wound status. **(B)** The continuous elevation of the score in the infection group was significant on day 6 and day 10. **(C)** The wound size in the infected mice was significantly increased on day 6 compared to the control group and was still trendwise enlarged on day 10 and day 13. **(D)** At least 2 different bacterial strains were determined in 86% of all wound infections and 57% of all infected wounds even showed at least 3 different strains of bacteria. Mean ± SD; P values: ** P ≤ 0.01, *P ≤ 0.05.

Scoring was performed based on 3D photos in the control and the infection groups infected with CS dilutions of 1:166–1:400. For each animal, each single parameter was scored and the sum of all scores (for each individual mouse) was composed into a heat map (Fig. 5A). Wound healing score of infected mice was consistently elevated by at least 20% over the entire observation period (days 1–13) compared to controls. The difference was statistically significant on day 6 and 10 (p ≤ 0.05; Fig. 5B).

The impact of infection on wound closure was confirmed by analysis of wound area, which was similarly increased in the infected group, showing a significant difference on day 6 and trendwise increased wound areas on day 10 and 13 (Fig. 5C).

Analysis of microbiological swabs (summarized for all CPA mice) provided a characterization of the bacterial wound phenotype (Fig. 5D). In 86% of the wounds at least two bacterial species were detected, in 57% at least three species.

**Figure 6:**
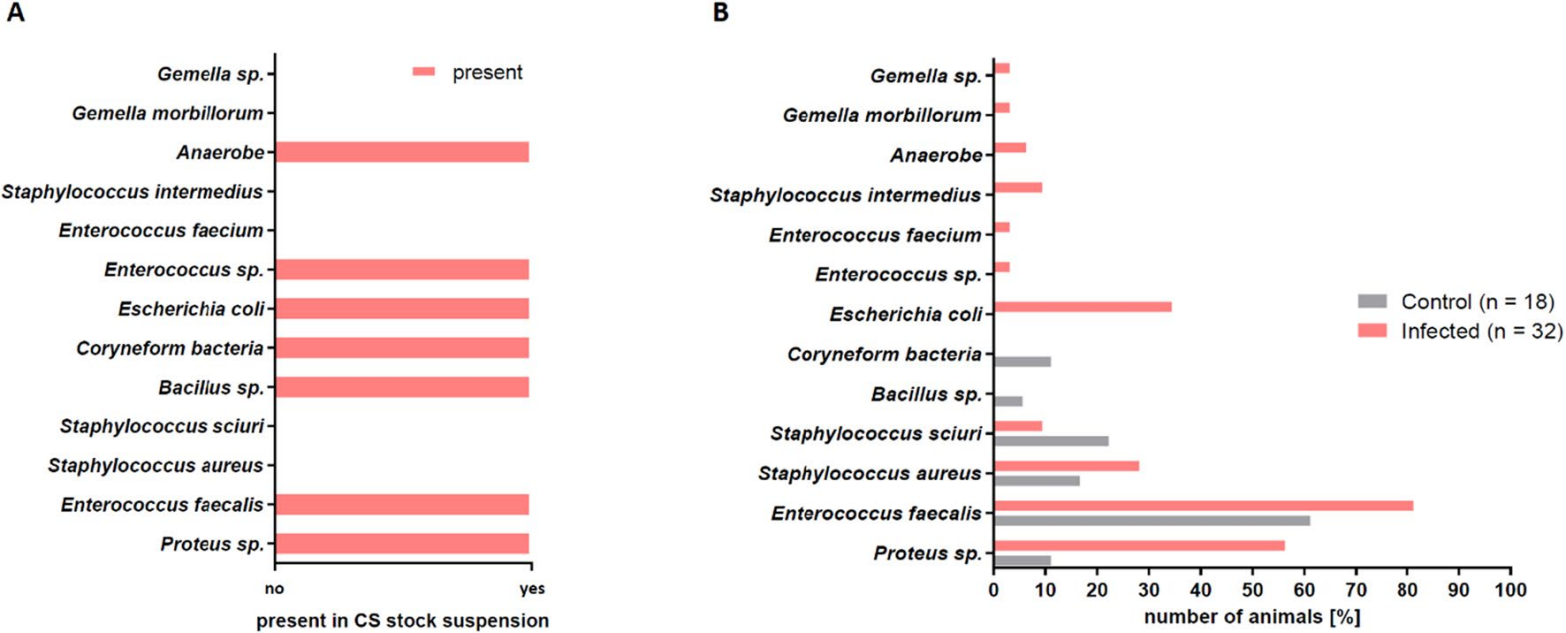
Comparison of the presence of different bacterial strains in the CS and the infected wounds. **(A)** Bacterial species detected in the CS analyzed before the infection procedure by microbiological swab analysis. **(B)** Bacterial species detected by microbiological swab analysis in the wound beds of control mice and wound beds of infected mice showed different profiles. Infected wounds showed a similar bacterial profile as the CS.

Figure 6 shows the comparison of bacterial species in the cecal slurry (Fig. 6A) and in the wounds (Fig. 6B) on day 3 post-surgery. Notable is the occurrence of Staphylococcus species in some wounds, which were not detected in the CS. These ubiquitous species also established in wounds of control animals despite standard wound disinfection.

Additionally, in order to provide detailed information and characterization of the content of the contaminating suspension, a metagenome analysis based on 3rd generation sequencing of CS was performed (Supplementary Fig. 1). A comparison of the phylogenetic tree with a catalog of the mouse gut genome^33^ showed a good accordance with the genera contained in our CS suspension (Supplementary Fig. 1A). The metagenome showed that bacteria constituted the largest fraction of about 55% of all reads assigned. Only a small fraction of 3.5% of bacterial genera of the CS were found in the wound beds analyzed by microbiological swab analysis, while among the others the anaerobic bacteria constituted a large fraction (Supplementary Fig. 1A). However, this method was not suited to be routinely used in this study.

#### Autofluorescence imaging of wound infections

**Figure 7:**
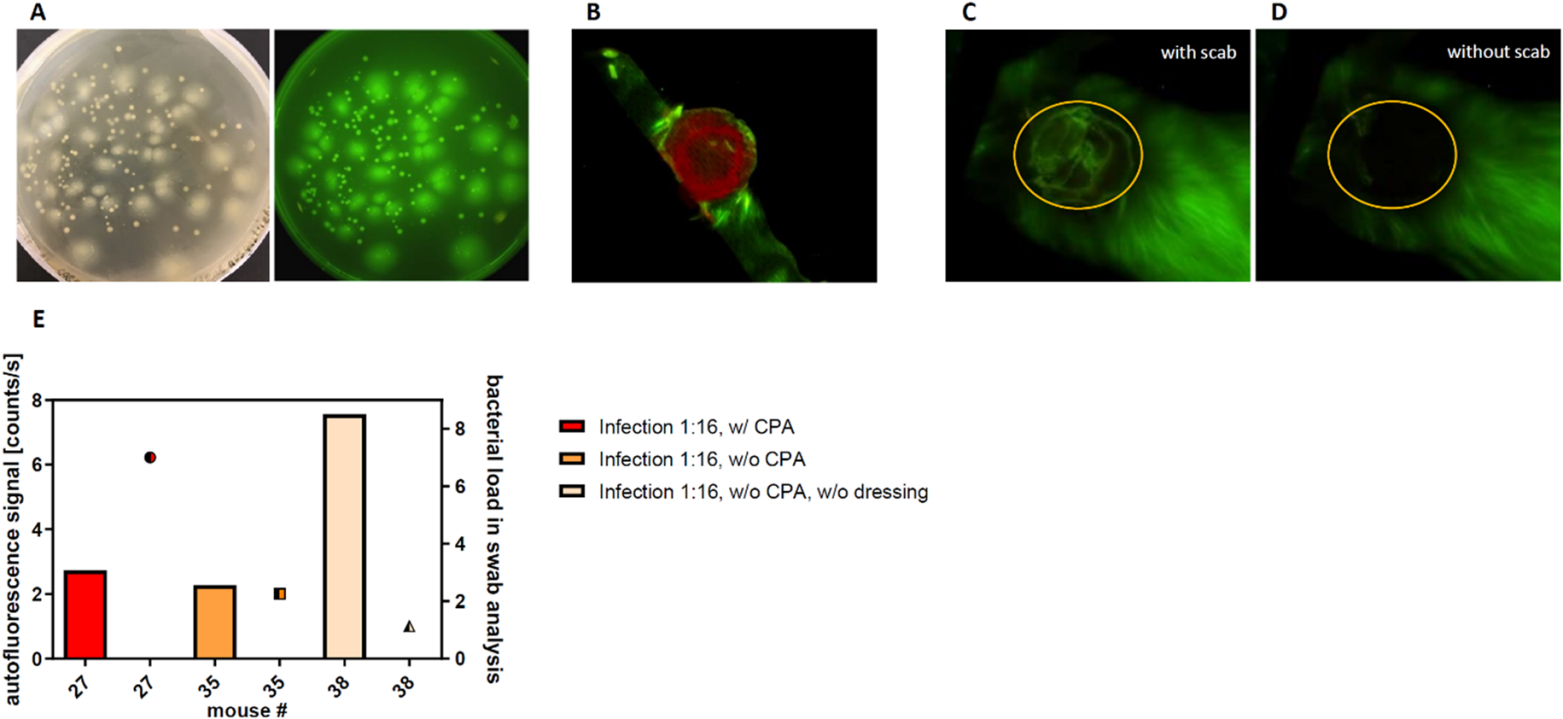
Autofluorescence tracking of wound infections. **(A)** Cecal slurry colonies showed a strong fluorescence signal in vitro. **(B)** By linear unmixing the wound exudate (red) and the autofluorescence signal of the fur (green) could be differentiated from the autofluorescence of the infection. **(C, D)** The scab formation strongly influenced the quantification of the infection autofluorescence. **(E)** The accordance of the quantified autofluorescence (bars) with the results of the microbiological wound swabs (dots) on day 3 post surgery was poor.

Recently, autofluorescence imaging was introduced to detect bacterial wound infections^34^. A secondary aim of this study was to test an in vivo fluorescence Maestro imaging system (CRi Inc., Woburn, Mass, USA) for the convenient tracking and quantification of the wound infections. First in vitro tests using CS smears on LB agar plates confirmed strong autofluorescence signal of all colonies visible on the plates (Fig. 7A).

However, in vivo data showed that the fluorescence signal was not specific enough for the detection of bacterial infection. The wound exudate, which was mainly observed on days 1–2 post-surgery, gave a strong positive signal, for example on the wound dressings shown in Fig. 7B. A longer follow-up time series over 7 days revealed that also scab formation significantly hampers the autofluorescence signal (Fig. 7C, D).

Accordingly, on day 3 post-surgery the autofluorescence signal (Fig. 7E, bars) of the wound did not correspond to the semi-quantitative results of the microbiological swab analysis (Figs. 7E, dots). For example, the wounds with the highest autofluorescence signal (Fig. 7E, light yellow bar) showed a low quantity in the microbiological swab analysis (Fig. 7E, light yellow dot).

## Discussion

In the setting of a compromised immune system, an exposure of fresh wounds to microbes may lead to severe infections and delayed wound healing^35^. Most of the currently existing wound models dealing with bacterial infections used either single pathogen species or combinations of selected bacterial strains^29,36–38^. However, under clinical conditions, patients are frequently confronted with complex wound infections, even though one strain might ultimately predominate. Wolcott et al. found that in a cohort of almost 3,000 chronic wound patients, 93% of the wound microbiomes were polymicrobial^39^. The authors investigated infections of ulcer wounds and detected a high proportion of *Staphylococcus* and *Pseudomonas* species in 63% and 25% of all wounds, respectively, but also observed a high prevalence of anaerobic bacteria and bacteria traditionally considered commensalistic. Similarly, Kalan and Brennan^40^ reviewed the role of the microbiome in nonhealing diabetic wounds and therein mentioned a study from Citron et al., which analyzed 454 diabetic foot ulcers and found a fraction of over 80% to be polymicrobial^41^. However, preclinical models simulating infections induced by a naturally occurring polymicrobial inoculum are missing. Therefore, this study aimed to establish a wound infection model in mice that includes the factor of naturally occurring microbial community interactions. For contamination a previously prepared and cryopreserved cecal slurry from feces of the same species was used. The choice of this methods was supported by a publication of Tipton et al. showing no significant influence in community composition after cryogenic preservation, whereas a significant loss of diversity was found when wound communities were transferred between species from human to mouse^42^. The complexity of this model provides the opportunity to subsequently test appropriate therapeutic approaches.

Various forms of immunodeficiency (existing as a congenital and/or acquired comorbidity) may constitute a serious impediment to the wound healing process given their poor tissue regenerative potential and/or low capacity for microbial clearance. For example, recipients of organ transplants^43^, and individuals suffering from human immunodeficiency virus/acquired immunodeficiency syndrome^44,45^ typically display an impaired wound healing^46^. A common feature of the native immune deficit includes an impaired infiltration/migration of granulocytes (predominantly neutrophils) to the infection site to clear the invading microorganisms^47^. Cyclophosphamide is a chemotherapeutic drug that is in clinical use for treatment of cancer and autoimmune diseases. Its administration induces neutropenia and lymphopenia, and therefore in the present study facilitated bacterial wound infections in mice by inhibiting the innate immune response^31,48,49^. Similar to other research groups, who studied single-strain infections^31,49^, we showed that immunosuppression with CPA is a necessary prerequisite for the establishment of severe polymicrobial wound infections using the CS inoculum. In contrast to some studies indicating a delayed wound healing after this pretreatment^23,50,51^, in the present study CPA by itself had no effect on the wound closure rate.

In pilot experiments, the infection with high CS doses (undiluted stock CS containing 1 × 10^9^/ml bacteria) in CPA-immunosuppressed mice resulted in clinical symptoms of severe systemic infection. These findings are in line with Thompson et al., who also faced septic events in a mouse model of wound infection with high doses of *A. baumanni*^52^. Our results showed that in accordance with Zululaga et al.^31^, who tested the same CPA dose in outbred ICR mice, the optimal CPA dose for reliable leukopenia in BALB/c mice was 250 mg/kg. Lower doses of CPA did not result in persistent signs of bacterial infection of the wound based on the wound healing score. However, CPA doses should be optimized for each individual infection model, animal species and strain to avoid systemic side effects. The dependence of infection-delayed wound closure on the CPA dose seemed to resemble rather an on–off-mechanism than a gradually configurable system. Therefore, fine-tuning of infection severity was performed in a second step by the gradual lowering of the CS inoculation dose. A similar process was previously reported in an immune-suppressed murine model infected with *Acinetobacter baumannii*, where sublethal doses (based on a normal immune system condition) of bacteria had to be reduced from 10^7^ CFU to 3.75 × 10^6^ CFU to lower the mortality rate from 100 to 20%^49^.

Besides the intended infection with known enterobacterial strains from the CS, some wound swabs showed additional contamination/infection with some ubiquitous microorganisms (i.e. predominantly *S. aureus*). This was problematic as *S. aureus* infections competed with bacterial strains from the CS inoculum and were able to overgrow them occasionally. The cooperative and competitive interactions of *S. aureus* with other bacterial strains and its altered behavior and increased persistency in polymicrobial communities were comprehensively reviewed by Nair et al.^53^ Thus, in the present study mice with infections solely showing ubiquitous bacterial strains that were not part of the CS inoculum based on the microbiological swab analysis were excluded.

In order to avoid auto-infections of the wounds with skin-resident bacterial flora, to achieve maximum difference between infected and non-infected wounds and to ensure optimal reproducible conditions, we decided to administer topical wound disinfection with Octenidindihydrochlorid (Octenisept). This recapitulates the current clinical practice given that wound disinfection has become a standard aim to prevent and manage inpatient wound infections as well as to reduce antibiotics and prevent development of antimicrobial resistance^54,55^. The presence/absence of the topical disinfection in control (uninfected) animals did not influence the wound closure speed (data not shown).

For basic research on bacterial infections, the use of luminescent bacterial strains and the detection by bioluminescence imaging are widely used tools^22,56,57^. However, as this procedure requires transiently transformed bacteria and in the present study we aimed to apply a complex biological sample (CS) for contamination, bioluminescence imaging was no option. Even so, tracking bioluminescence signals of transiently transformed bacteria in long-term in vivo studies is rather impossible due to two main reasons: (a) an comparable transformation with similar bioluminescent signal cannot be reached for all bacterial species in biological samples, like CS, (b) stable expression and a reliable quantitative signal could not be guaranteed since the selective pressure cannot be maintained over several days. Therefore, we aimed to establish a multifactorial analysis tool to assess the impact of feces-induced infections on wound healing including microbiological swab analysis, wound closure measurement, a newly established wound score and also tested autofluorescence imaging.

Real-time monitoring of wound infections by autofluorescence imaging is a tool for clinical diagnostics. Both preclinical^34^ and clinical^58,59^ studies showed that autofluorescence-guided sampling confirmed the proper choice of antibacterial treatment strategies. In the present study a similar approach was tested using in vivo autofluorescence imaging for the follow-up detection of feces-induced infections. The Maestro imaging device allows to analyze autofluorescence in anaesthetized rodents. In vitro*,* the signal of the cultivated CS-derived colonies was strong and adequately defined. However, in vivo the autofluorescence signal strength did not correspond with the results of the standard microbiological cultures. The authors assume that the bacterial load in the wound was not high enough for reliable detection given that devices for clinical use are reported to be best for the detection of moderate to heavy bacterial growth^59^. Since the early assessment of infections is crucial for preventive treatment approaches, we refrained from further use of autofluorescence imaging in this model setup. Thus, we supplemented the quantitative analysis of wound closure and microbiological swab analysis with a qualitative analysis using a wound healing score. We modified previously published wound scores^60,61^ to describe the differences observed between control and infected wounds by assigning them to five different categories with a four-point scale. The summarized score was continuously significantly increased in the infection group. Eventually the comprehensive wound evaluation, including wound closure analysis, microbiological swab analysis and the wound healing score, enabled a reliable differentiation between control and infection group.

In conclusion, in this study we present the establishment of a simple and reliable in vivo skin wound model, infected with a naturally occurring bacterial suspension. The combination of quantitative and qualitative wound analyses guarantees a comprehensive evaluation of the infection and wound healing progress. Therefore, this model can be used for the testing of infection-control therapies as well as strategies to improve infection-impaired wound healing and thereby drive the supporting and/or alternative treatment approaches to prepare for the post-antibiotic era.

## Supplementary information

Supplementary Information.
