# Supplementary material for: Contamination of wounds with fecal bacteria in immuno-suppressed mice
Source: Sci Rep. 2020 Jul 13;10:11494. doi: 10.1038/s41598-020-68323-5 (PMC7359036; doi:10.1038/s41598-020-68323-5)
Supplement: Supplementary file 1 — Supplementary Information. [file 41598_2020_68323_MOESM1_ESM.docx]

**Supplementary Material**

**Contamination of wounds with fecal bacteria in immuno-suppressed mice**

Lisa Karner^1^, Susanne Drechsler^1^, Magdalena Metzger^1^, Paul Slezak^1^, Johannes Zipperle^1^, Guadalupe Pinar², Katja Sterflinger², Friedrich Leisch³, Johannes Grillari^1^, Marcin Osuchowski^1^, Peter Dungel^1,^*

^1^ Ludwig Boltzmann Institute for Experimental and Clinical Traumatology, Vienna, Austria

^2^ Department of Biotechnology, University of Natural Resources and Life Sciences, Vienna, Austria

^3^ Institute of Statistics, University of Natural Resources and Life Sciences, Vienna, Austria

*Corresponding author:

Peter Dungel

Ludwig Boltzmann Institute for Experimental and Clinical Traumatology in the AUVA Research Center

Donaueschingenstraße 13

A-1200 Vienna, Austria

Tel.: +43 59393 41972

E-mail: [peter.dungel@trauma.lbg.ac.at](mailto:peter.dungel@trauma.lbg.ac.at)

Supplementary Figure 1


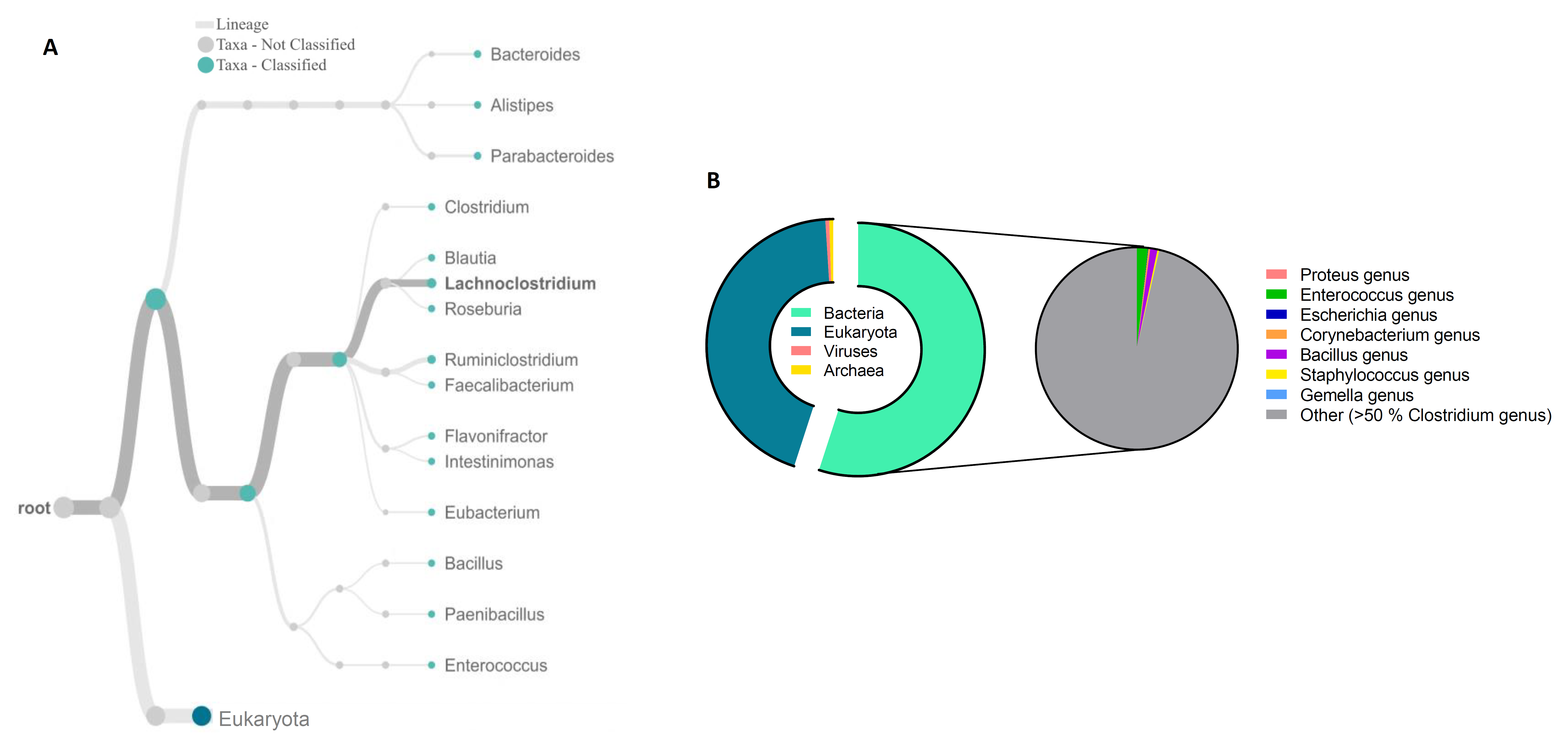


Suppl. 1: Analysis of the CS suspension by 3^rd^ generation sequencing.

(A) Tree representation of the taxonomical assignment of the sequenced reads, at a cutoff of 0.5 % and the genus level, delivered by the cloud-based data analysis platform Epi2me *(Desktop Agent v2019.7.9)* using the WIMP workflow FastqWIPM-v2.1.1 (Oxford Nanopore technologies) (https://epi2me.nanoporetech.com). The branch thickness is proportional to the share of reads assigned to the taxonomy. (B) Out of the bacterial fraction (55 %), about 3.5 % showed genera found in the microbiological analysis of the wound swabs taken from the wound beds. The largest group accounts for anaerobic bacteria.
